# Nurses' Psychosocial Barriers to Suicide Risk Management

**DOI:** 10.1155/2011/650765

**Published:** 2011-06-01

**Authors:** Sharon Valente

**Affiliations:** Research and Education, Department of Veterans Affairs, 11301 Wilshire Boulevard, Room 6235, Los Angeles, CA 90073, USA

## Abstract

Suicide remains a serious health care problem and a sentinel event tracked by The Joint Commission. Nurses are pivotal in evaluating risk and preventing suicide. Analysis of nurses' barriers to risk management may lead to interventions to improve management of suicidal patients. These data emerged from a random survey of 454 oncology nurses' attitudes, knowledge of suicide, and justifications for euthanasia. Instruments included a vignette of a suicidal patient and a suicide attitude questionnaire. *Results*. Psychological factors (emotions, unresolved grief, communication, and negative judgments about suicide) complicate the nurse's assessment and treatment of suicidal patients. Some nurses (*n* = 122) indicated that euthanasia was never justified and 11 were unsure of justifications and evaluated each case on its merits. Justifications for euthanasia included poor symptom control, poor quality of life, incurable illness or permanent disability, terminal illness, and terminal illness with inadequate symptom control or impending death, patient autonomy, and clinical organ death. The nurses indicated some confusion and misconceptions about definitions and examples of euthanasia, assisted suicide, and double effect. Strategies for interdisciplinary clinical intervention are suggested to identify and resolve these psychosocial barriers.

## 1. Psychosocial Barriers to Suicide Risk Management

Patients facing a life-threatening illness such as cancer have an increased risk of suicide, and this study examines the nurse's psychosocial barriers to managing suicide risk. Nurses have a major role to play in patient safety when they recognize the warning signs, monitor the patient's emotional state, provide a therapeutic relationship, and take precautions to prevent suicide. Although 70% of people warn providers of their suicidal impulses, clinicians often fail to take these warnings seriously [1]. Therapeutic intervention can often effectively help alleviate the pain, symptoms, or depression and reduce suicide risk. Psychosocial barriers such as the nurse's emotions, beliefs, knowledge, or attitudes can impair risk management. This paper describes content analysis of oncology nurses' narratives about psychosocial barriers in managing suicide risk. 

People with cancer have higher than average rates of suicide. Rates of suicide have been estimated to be as high as 31.4/100,000 person-years among people with cancer or AIDS. [2]. Misono et al. found an age-, sex-, and race-adjusted rate of 31.4/100,000 person-years which is almost twice the general suicide rate in the US which was 16.7/100,000 person-years. Men who were older and white had higher suicide rates. Specific cancers had the highest suicide risks (e.g., lung and bronchi cancers (standardized mortality ratio (SMR) = 5.74; 95% CI, 5.30 to 6.22), stomach (SMR = 4.68; 95% CI, 3.81 to 5.70), oral cavity and pharynx (SMR = 3.66; 95% CI, 3.16 to 4.22), and larynx (SMR = 2.83; 95% CI, 2.31 to 3.44). Suicide is a sentinel event. Suicide risk increases when cancer patients face diagnosis, disease exacerbation, treatment failure, and advanced or terminal stage of illness. An estimated 2–6% and an average of 3% of terminally ill cancer patients commit suicide or request assistance to do so [3]. A person's threats of suicide may mean that the person wants to escape from pain, fears, and misery and they may consider suicide the only option. However, therapeutic intervention can resolve some of the painful and distressing symptoms.

## 2. Purpose

This paper reports the analysis of the psychosocial factors (e.g., emotions, personal experiences, values, and judgments) that oncology nurses identified as barriers to their management of suicide risk of a suicidal patient depicted in a vignette. These data emerged from a study that examined the oncology nurse's attitudes and knowledge in managing suicide risk.

## 3. Methods

Four hundred and fifty-four oncology nurses (37%) from a national, random survey of 1200 Oncology Nursing Society members in clinical practice provided informed consent and agreed to participate. Clinical oncology nurses were selected because they cared for patients with increased risk of suicide. Six retired nurses declined to participate.

## 4. Instruments

Instruments included a demographic inventory, a vignette of a suicidal patient with questions about nursing evaluation and management, a quantitative instrument with 94 items measuring attitudes toward suicide of the self, a loved one and a stranger in various situations, and a Suicide Attitude Questionnaire, a qualitative tool. The instruments were pilot tested, revised, and given in random order to participants. Participants used a confidential code and mailed back the questionnaires.

## 5. Suicide Vignette

 A case study of an oncology patient with suicidal signs (e.g., older, Caucasian, widower, depressed, talks about suicide, gives away prized possessions, and has no reason to live) was presented. It included questions about evaluating risk assessment, evaluation, psychosocial assessments, suicide risk factors, depression and anxiety ratings, goals and interventions, skill, and knowledge. In the development of these tools, both content validation and interrater reliability were assessed. The procedure employing content experts for content validation followed the usual protocol. A judge panel of experts with advanced training in psychosocial oncology and psychiatric nursing established content validity and scoring. Test-retest (94%) was measured and inter-rater reliability (96%) in scoring and data entry was accomplished by having two people rate more than 25% of the vignettes. 

Vignettes simulate a real situation and provide an effective research tool to elicit respondent's attitudes, knowledge, opinions, interventions, and respondent's anticipated behavior in the situation [4]. Outcomes assessment in psychiatric postgraduate medical education: an exploratory study using clinical case vignettes [4, 5], (Russell, 2007). Vignettes can collect information simultaneously from a large sample, manipulate variables, and avoid ethical problems that might occur in observational studies [4]. Vignettes have served to elicit diagnoses [6], pain [7], nursing knowledge [8], nursing performance [9], infection control [10], ethical decisions [11], and schizophrenia [12]. Expert panels have been used to validate vignettes. 

The Suicide Attitude Questionnaire (SAQ) is a qualitative measure with items about care giving, ethical issues, knowledge and assessment, and open-ended questions about the difficulty responding to a patient who mentions suicide, circumstances that would justify euthanasia, circumstances under which a patient's request for assistance should be granted, concerns and conflicts, and questions about suicide knowledge and suicide assessment skill. Questions about euthanasia were added because the pilot study showed that nurses were confused about this issue and needed an opportunity to express these issues. Nurses answered these questions and rated their skill and knowledge in suicide assessment and management. Psychometrics for the instruments are described in detail elsewhere [13].

## 6. Qualitative Data Analyses

Content analysis afforded a systematic approach for analyzing narrative texts into categories and making sense of the data. All written narrative responses to SAQ items were entered verbatim for each question and typed into Word, a word processing program. We followed Wilson's (Wilson, 1989) procedures for semantic content analysis. We identified the units of analysis as the respondent's words. Our unit of analysis were words (e.g., fear, not difficult), phrases (e.g., fear of reprisal by family, fear of failure), or sentences (e.g., due to chronic illness, my parent committed suicide) that conveyed a unified idea. Six responses were not able to be coded because they were vague or unclear. 

The principle investigators trained the data analysis team (e.g., experienced masters-prepared oncology nurses, an oncology social worker, and a graduate nursing student) in qualitative data analysis. The doctorally prepared principal investigators had expertise in psychosocial oncology. After the narrative data were entered in the computer and verified, the data analysis team independently read the narratives before meeting to discuss and identify categories. We developed the preliminary categories from the data, rationale, and illustrations to guide the coding. The entire research team met to refine the directions for coding until categories were accepted for coding the narratives. After describing each category for coding, the research team independently assigned the written narrative responses to these categories before meeting to discuss our coding and reach consensus. Final decisions about coding were reached by consensus through discussion. The following categories emerged: religious, spiritual, or other values and beliefs; uncomfortable feelings; personal experiences with suicide; inadequate skills, knowledge and experience (in suicide evaluation and treatment); weight of professional responsibility; not difficult to care for suicidal patients.

## 7. Results

### 7.1. Demographics

Of the total sample, 90.4% were women. Seventy-seven percent (77%) were EuroAmerican, 7.1% were African Americans, 7.1% were Asian Americans, 4.2% were Latin Americans, and 2.1% identified themselves as Canadians. The modal age was 40–49 with a range of 20 to over 60 years old. Most of the sample had a diploma/AA or B.S. degree (38.4%) or an M.S. degree (55.9%). Most nurses (89.1%) had worked more than 9 years in nursing. Although most respondents were educated in the United States (91.8%), some were educated in Canada or abroad. We aimed for a sample of nurses in clinical practice. Most nurses reported spending at least 50% per day in clinical practice. Nearly 90% of nurses were close to one or more patients (*x* = 6 who died in the year preceding the survey). Nearly all nurses had taken courses in cancer; approximately 20% had taken a course in suicide. 

The main categories or core concepts that emerged from the interviews and focus group included communication barriers, judgments about suicide, unresolved grief, emotions, inadequate knowledge, and justifications for euthanasia.

### 7.2. Communication Barriers

They emerged from the nurses' narratives about their difficulties caring for a suicidal patient. Some nurses explained that they lacked the expertise of psychiatrists and did not know what to say to a suicidal patient (1) “I would say nothing to avoid making an error.” Nurses also indicated they lacked skill and knowledge about suicide risk assessment. (2) “I would not know what to ask about suicide so I remain silent.” (3) Others feared that asking about suicide risk might encourage suicidal acts. Some nurses also reported that they did not know how to tell if the patient was “serious” about these suicidal impulses and did nothing while trying to figure out if the patient was serious. Some nurses expressed conflicts between their roles in suicide prevention and advocacy for the patient at end of life who wanted to die with dignity. One nurse said, “I knew I should sound the alarm when the patient talked about suicide but he was dying and was exhausted by the treatments and wanted to die in peace.” 

### 7.3. Judgements about Suicide

Nurses reported that suicide was “a coward's way out” and some nurses identified religion as the source of their values, but others described spiritual or other beliefs that led them to judge suicide as wrong or bad. Nurses expressed concern about what was morally right for the patient within the limits of what was required by the legal and professional guidelines. Some nurses worried about the impact on the family and thought the patient was wrong for harming the family. Examples of nurse's narratives include the following: (1) “I was reared with a strong religious belief that ending one's life should be prevented” and (2) “Topic is taboo in society; my religious conviction—suicide is wrong—a mortal sin.”

### 7.4. Unresolved Grief

Nurses drew on experience or lack of experience in their own family or personal life and shared personal experiences. When a family member had committed suicide several nurses said that their reaction to a family member's suicide impaired their ability to care for suicidal patients. Examples of nurses' narratives included the following: (1) “My grandmother committed suicide and I had an uncle too.” (2) “A close relative of mine did (committed suicide) for health reasons. Its very sad.” 

### 7.5. Emotions

Nurses described unpleasant emotional responses of varying or unspecified intensity (e.g., related to fear and other feelings) that created difficulty. Examples of nurses' narratives include the following: (1) “Fear they will commit suicide. I always think about the pain they must be in and the level of that despair. Suffering is hard to watch.” (2) “Fear of reprisal by patient/family.” (3) “Very uncomfortable feeling.” Other comments included “fear that I'm unable to help and the feeling that I'm a personal failure if the patient commits suicide.” Some nurses reported “I'm just uncomfortable with the subject.” 

*Inadequate Knowledge*. I do not know the right thing to say. Nurses highlighted deficits in their professional experience, skill, knowledge, or abilities to care for suicidal patients. The narrative was cognitive in tone and description. Some nurses said that they lacked the skill and knowledge to work with suicidal patients. They did not know how to respond and wanted to avoid the risk of responding incorrectly. In this category, respondents focused on suicide. Examples from the nurses' narratives included the following: (1) “Sense of failure that I might not be able to “fix” things. Its frightening; I'm not skilled at assessment and counseling in this situation. Uncertainty about my own skill/knowledge related to assessment.” (2) “Inadequate and ambivalent; unfamiliar with suicide; no education.” (3) “Not knowing how to help their helplessness, finding a way to reach them and get them to see suicide as absolutely the last alternative.” (4) “I don't want to say the wrong thing.”

## 8. Circumstances That Justify Euthanasia

Asking about suicide in the context of end of life raises issues about euthanasia. Nurses struggle to weigh patient rights with their personal values and professional beliefs about what is right. This is never an easy decision, as nurses see patients suffer at end of life needlessly and those who have intrusive interventions despite their wishes to the contrary as well as patients who want to let nature take its course. Nurses reported the circumstances that would justify euthanasia. For nurses (*n* = 122) euthanasia was never justified. Other nurses indicated that euthanasia could be justified for poor symptom control or quality of life (not terminally ill) (*N* = 61), terminal illness, impending death with qualifiers (pain, QOL, end-stage disease) (*N* = 69); patient autonomy (informed choice, living will) *n* = 30; incurable illness/permanent disability *n* = 23; clinical death, organ donor, vegetative state, flat EEG *N* = 15; terminal illness (no qualifier) *n* = 14; not sure *n* = 11.

## 9. Discussion

The nurse's concerns related directly to complex professional and ethical issues that were embedded in the various contexts of nursing care. Clinicians reported conflicts as they considered their duties to safeguard life and to respect the patient's autonomy, yet they did not report considering the patient's capacity to exercise autonomy. Some believed that their duty was to respect a patient's right and freewill to choose suicide instead of a life-threatening disease (e.g., cancer or AIDS) that involved pain, fatigue, and unrelieved suffering. On the other hand, nurses had a duty to protect patients from harm. 

Some nurses may not thoroughly consider the criteria for rational thinking and voluntary, informed choice that is necessary for autonomy. In some instances, suicidal patients lack capacity to exercise autonomy because a severe psychiatric disorder such as major depression with cognitive deficit undermines their ability to think clearly. However, the striking fact is that nurses did not describe evaluating rational thinking in their consideration of what justified euthanasia. 

Nurses rarely emphasized the constraints of their settings. They struggled to describe their responses and actions in the context of moral versions of the responsible nursing role in situations where the nurses were not the arbiters of power or decisions. In attempting to serve the best interests of all involved, nurses face difficult ethical dilemmas. The nurse's strong support for the ethical principles of autonomy, or free choice, and self-determination may conflict with nonmaleficence, or the duty to prevent harm. When a patient asks for the nurse's assistance in dying, the nurse must consider the conflicting ethical duties to prevent harm and respect the patient's choice. The ICN Code of Ethics states that “the nurse takes appropriate action to safeguard the individual when his care is endangered by any other person” (ICN, p. XIII). In addition the nurse needs to consider responsibilities to the family, hospital. Professional, legal, and ethical implications arise when terminally ill patients request euthanasia.
